# Fiber Optic pH Sensor with Self-Assembled Polymer Multilayer Nanocoatings

**DOI:** 10.3390/s130201425

**Published:** 2013-01-24

**Authors:** Li-Yang Shao, Ming-Jie Yin, Hwa-Yaw Tam, Jacques Albert

**Affiliations:** 1 Institute of Optoelectronic Technology, China Jiliang University, Hangzhou 310018, China; 2 Department of Electrical Engineering, The Hong Kong Polytechnic University, Hung Hom, Kowloon, Hong Kong; E-Mails: ming-jie.yin@connect.polyu.hk (M.-J.Y.); eehytam@polyu.edu.hk (H.-Y.T.); 3 Department of Electronics, Carleton University, Ottawa, ON K1S 5B6, Canada; E-Mail: Jacques_Albert@carleton.edu.ca

**Keywords:** fiber-optic sensor, layer-by-layer self-assembly, multilayer film, pH sensor, tilted fiber Bragg grating

## Abstract

A fiber-optic pH sensor based on a tilted fiber Bragg grating (TFBG) with electrostatic self-assembly multilayer sensing film is presented. The pH sensitive polymeric film, poly(diallyldimethylammonium chloride) (PDDA) and poly(acrylic acid) (PAA) was deposited on the circumference of the TFBG with the layer-by-layer (LbL) electrostatic self-assembly technique. The PDDA/PAA film exhibits a reduction in refractive index by swelling in different pH solutions. This effect results in wavelength shifts and transmission changes in the spectrum of the TFBG. The peak amplitude of the dominant spectral fringes over a certain window of the transmission spectrum, obtained by FFT analysis, has a near-linear pH sensitivity of 117 arbitrary unit (a.u.)/pH unit and an accuracy of ±1 a.u. (in the range of pH 4.66 to pH 6.02). The thickness and surface morphology of the sensing multilayer film were characterized to investigate their effects on the sensor's performance. The dynamic response of the sensor also has been studied (10 s rise time and 18 s fall time for a sensor with six bilayers of PDDA/PAA).

## 1. Introduction

pH monitoring is widely demanded in biomedical, environmental, and industrial fields such as clinical blood analysis, industrial wastewater treating, food processing, and process control in bioreactors [1]. However, the conventional pH electrodes are always too voluminous to be utilized for *in vivo* pH measurements, especially in medicine and clinical analysis [2]. Compared with electrical pH sensors, optical fiber-based pH sensors exhibit many advantages such as compact size, electrically passive operation, immunity to electromagnetic interference, capability of remote measurement and multiplexed detections [3,4]. Different fiber-optic pH sensors have been demonstrated by depositing a pH-sensitive coating onto the fiber surface such as hydrogels [5], cellulosic films [6], sol-gel polymers [7]. Recently, layer-by-layer (LbL) electrostatic self-assembly (ESA) technique has emerged as a versatile and convenient method for preparing ultrathin multilayer films due to their precise control of thickness at the nanometer scale and deposition on non-flat surfaces [8–11]. Goicoechea demonstrated a Fabry-Perot (FP) nanocavity pH sensor by coating the nanostructured film on the fiber-end surface and white-light interferometry is employed for the measurement of pH-induced swelling of the film [12]. Corres presented a long period grating (LPG) pH sensor where the nanocoating was deposited on the circumference of the LPG as the reacting functional film and the resonant wavelength shift of the LPG was utilized for the determination of pH-induced refractive index (RI) changes [13]. Gu proposed a simple fiber-optic pH sensor based on the thin-core fiber modal interferometer (TCFMI) [2]. The LPG and TCFMI sensors have similar problems in locating the exact resonant wavelength because of their large bandwidths. The TCFMI scheme also has the disadvantage of high insertion loss due to the mismatch of fiber core diameter, which requires a relatively high power light source for reasonable signal to noise ratio and might limit its multiplexity ability.

In this paper, we propose a new fiber-optic pH sensor based on a TFBG with PDDA/PAA multilayer sensing films. The PDDA/PAA coating has a high refractive index (relative to the material of the fiber, *i.e.*, silica), so not only the resonance wavelength but also the coupling strength change with the pH of the solution. We will show that the visibility of the transmission spectrum oscillations caused by cladding mode resonances increases with the pH. Similar to our work on a TFBG pressure sensor [14], by applying a fast Fourier transform (FFT) analysis of a suitable region of the transmission spectrum, we obtain a direct measure of the visibility and hence of the pH of the solution. Another advantage of the TFBG pH sensor is its inherent temperature reference that is provided by the core mode reflection wavelength. Finally, dynamic measurements show that fast responses were obtained with a rise time (t_r_) of 10 s and fall time (t_f_) of 18 s for the sensor with six bilayers of PDDA/PAA.

## 2. Experimental Procedures

### 2.1. Materials

Poly(diallyldimethylammonium chloride) (PDDA, Mw = 100,000∼200,000 g·mol^−1^, 20% aqueous solution, refractive index: 1.375), and poly(acrylic acid) (PAA, Mw = 100,000 g·mol^−1^, 35% aqueous solution, refractive index: 1.393) were purchased from Aldrich (St. Louis, MO, USA). Hydrochloric acid (HCl) and sodium hydroxide (NaOH) were all analytical reagents. Deionized water with a resistance of 18 MΩ cm was used in all experiments.

### 2.2. Preparation of (PDDA/PAA)_n_ Multilayer Films

The preparation process is similar with our previous works [3,15,16]. Both PDDA and PAA solutions were prepared with a concentration of 2.0 g·L^−1^ and a pH of 3.0. The pH of all solutions was checked with a digital precision pH meter (model PHS-5E, Changhong Ke Ji, Shenzhen, China), with an accuracy of ±0.01 pH. The LbL electrostatic self-assembly of oppositely charged polyelectrolytes (positively charged PDDA and negatively charged PAA) was carried out on the substrates (here they were AT-cut quartz crystals with gold electrode, quartz slides (10 × 20 mm^2^) and TFBG. The substrates were cleaned by piranha solution (8:2 of 95% H_2_SO_4_ and 50% H_2_O_2_), washed with large amounts of deionized water and dried with nitrogen, obtaining negatively charged substrates. The negatively charged substrates were dipped into the positively charged PDDA and negatively charged PAA solutions alternatively, each for 10 min at room temperature. Between polycation and polyanion immersions, the substrates were rinsed in deionized water three times (1 min each time) to remove any excess of adsorbed material and dried with nitrogen. A coating made by two dipping procedures was called a bilayer, named as (PDDA/PAA)_1_. The same cycle was repeated until the desired number of bilayers was deposited.

### 2.3. Thin Film Characterizations

The fabrication process and thickness of the multilayer film were traced by quartz crystal microbalance measurements (QCM, Resonance Probe GmbH, Goslar, Germany). AT-cut quartz crystals (Maxtek, Shanghai, China) with a fundamental frequency *f_0_* of 5 MHz and gold electrodes were used. The multilayer films were fabricated on the front side of the quartz crystal. The frequency shift of the quartz crystal, *Δf*, after the deposition of each layer was measured at three overtone orders n = 1, 3, 5 (*i.e.* 5, 15, and 25 MHz) and the corresponding thickness was estimated with the Sauerbrey equation with the assumption that the density of the multilayer film is 1.0 g·cm^−3^ [15] in Equation (1):

(1) $$d_{f}=-\frac{Z_{q}\cdot \Delta f}{2f_{0}\cdot f\cdot \rho_{f}}=(-5.87\times 10^{-2})\Delta f(nm)$$

where Δ*f* is frequency shift, *f* = n*f*_0_ is the frequency, *ρ_f_* is the density of the film, and *Z_q_* = 8.8 × 10^6^ kg·m^−2^·s^−1^ is the acoustic impedance of crystalline quartz.

Atomic force microscopy measurements (AFM, tapping mode) were performed by using a Seiko SPI3800N station (Seiko Instruments Inc., Tokyo, Japan). Silicon tips (NSG10, NT-MDT) with a resonance frequency of *ca*. 330 kHz were used.

### 2.4. Grating Fabrication

Corning SMF-28 fiber was used to fabricate the TFBGs. The fiber was soaked in a hydrogen chamber for 12 days at 2,500 psi and room temperature of 25 °C to improve its photosensitivity. 1-cm-long TFBG with a tilt angle of 10° was inscribed in the hydrogen-loaded fibers using a pulsed KrF excimer laser and the phase mask technique. To excite high order cladding modes spectrally located in the C+L band (1,520–1,610 nm), the period of phase mask was chosen to be 1,106.5 nm. The resulting Bragg wavelength of TFBG is 1,602.36 nm. A broadband light source and optical spectrum analyzer were used to measure the spectrum evolution of the sensing TFBG. Another optical spectrum analyzer with sampling rate of 1 kHz and bandwidth from 1,510 nm to 1,590 nm (from Micron Optics, Inc., Atlanta, GA, USA) was utilized for the dynamic measurement.

## 3. Results and Discussion

**Figure 1.**
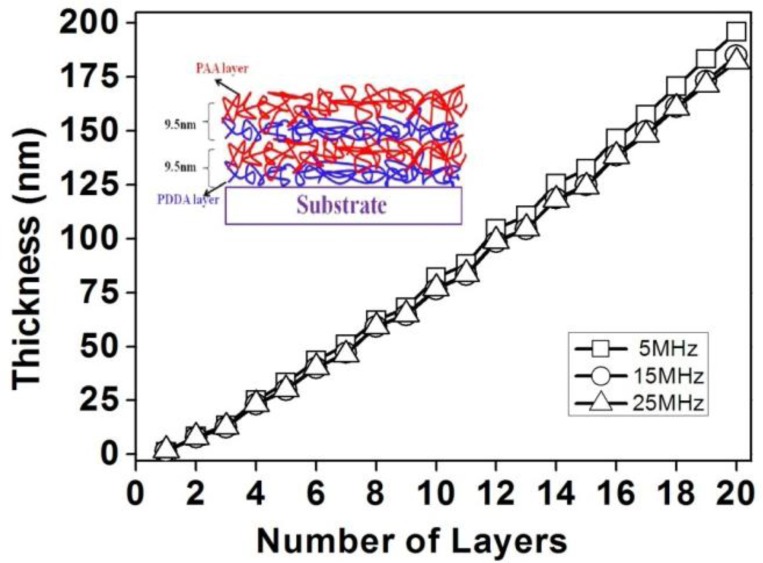
Thickness measurement with different number of bilayers. The insert shows the schematic diagram of LbL electrostatic self-assembly multilayers.

The PDDA/PAA multilayer films were firstly deposited on AT-cut quartz crystals to study the LbL assembly process using QCM. Figure 1 shows that the thickness of multilayer films grows almost linearly with increasing layer numbers. this indicates uniform film stacking by the LbL method [3,17]. The thickness of (PDDA/PAA)_10_ multilayer film is about 185 nm, which means about 9 nm for each layer thickness. Because of this stackability, the thickness of the multilayer films fabricated by LbL can be controlled to within a few nanometers. This control is very important for preparing fiber-optic sensors, as some types of sensors have a very short range of penetration of the optical probing field outside of the fiber [4].

**Figure 2.**
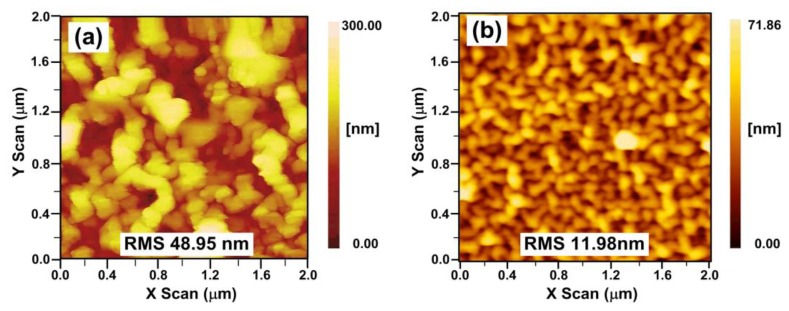
AFM images of multilayer nanocoatings (***a***) (PDDA/PAA)_4_ and (***b***) (PDDA/PAA)_10_.

Figure 2 gives the atomic force microscopy (AFM) images of (PDDA/PAA)_4_ and (PDDA/PAA)_10_ multilayer films (dry). A wormlike or vermiculate pattern can be observed for the multilayer, which is a general phenomenon for common polyelectrolyte multilayer films [3,18]. However, comparing Figure 2(a,b), the morphology of the (PDDA/PAA)_4_ multilayer film has smaller, less connected aggregates and larger root mean square (RMS) height, that is 48.95 nm, while for the (PDDA/PAA)_10_ multilayer film, larger clusters are formed that tend to connect together, with a smaller RMS of 11.98 nm. The reason is that as the layer number increases, the polyelectrolyte chains will fill up the surface voids of previous multilayer film [19].

**Figure 3.**
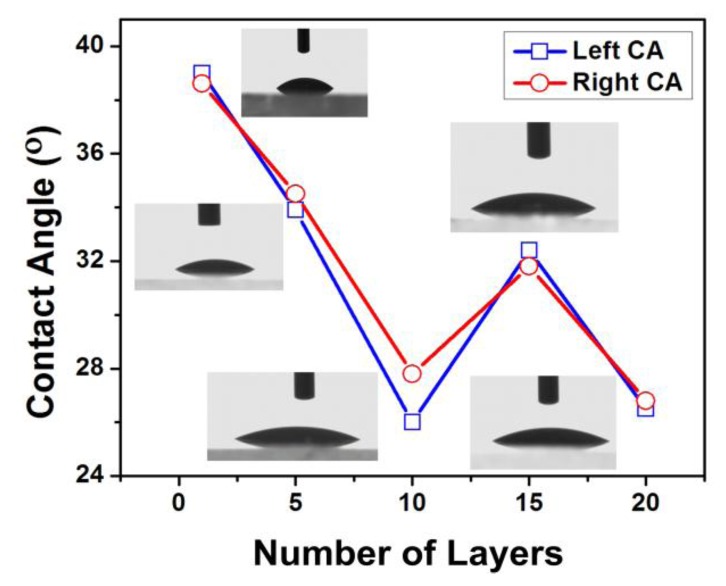
Water contact angles (CA) for PDDA/PAA multilayer films.

We also tested the water contact angles (CA) for PDDA/PAA multilayer films. Figure 3 shows how the CA changes with the number of layers. It can be seen that the CA for PDDA as the outermost layer is about 30° while with PAA as the outermost layer it is about 25°, which means a strong hydrophilic property of the sensing film, so the PDDA/PAA multilayer films are suitable for pH sensing as the test is in water solutions.

**Figure 4.**
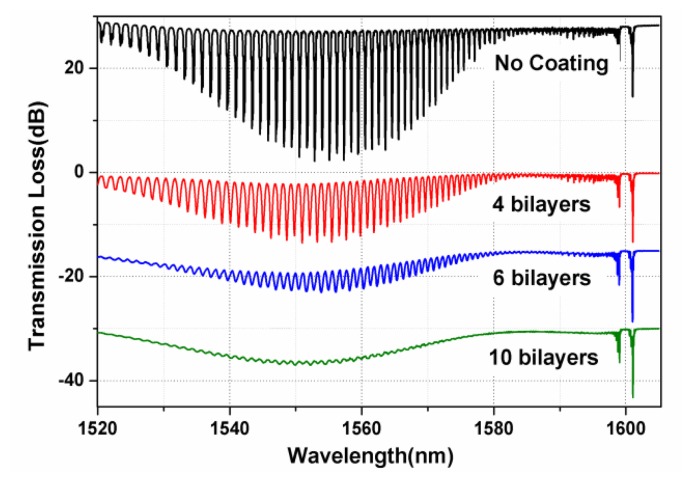
Transmission spectra of 10° TFBG without coating and with 4, 6 and 10 bilayers [(PDDA/PAA)_4_, (PDDA/PAA)_6_, (PDDA/PAA)_10_].

Figure 4 shows the transmission spectra of a 10° TFBG without coating and with 4, 6 and 10 bilayers of PDDA/PAA multilayer sensing film. When the sensing film gets thicker, the high-order cladding mode resonances are largely suppressed and a broadband radiation loss exists due to the coupling from the core mode to the radiation mode continuum. That is because when the RI of surrounding medium increases to or over that of the fiber cladding, the coupling strength of higher-order cladding mode decreases strongly. Figure 4 also further demonstrates that our PDDA/PAA multilayer sensing film have been successfully deposited on the surface of the TFBG.

**Figure 5.**
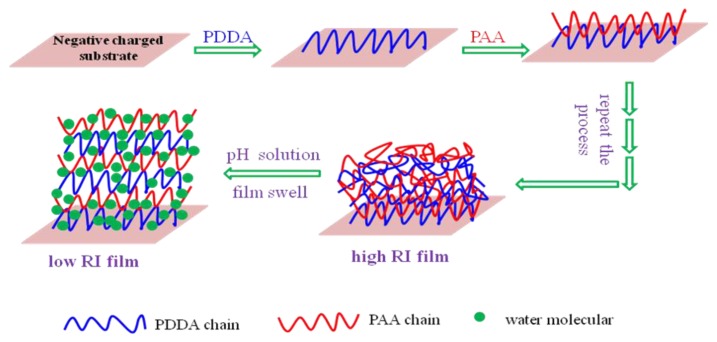
Schematic principle of TFBG based pH sensor.

The fabricated TFBG pH sensor was packaged into a shallow alumimum box with two ends of grating fixed to avoid the bend of fiber affecting on the results of pH measurement. Three sensors with (PDDA/PAA)_4_, (PDDA/PAA)_6_, (PDDA/PAA)_10_ multilayer sensing film have been packaged and tested one by one. The principle of our TFBG based pH sensor is shown in Figure 5. As the swelling state of the PDDA/PAA multilayer sensing film changes with the pH of the solution, the RI of the sensing film will also change accordingly [11,20]. As a result, the transmission spectra of the fiber-optic sensors will also be impacted.

**Figure 6.**
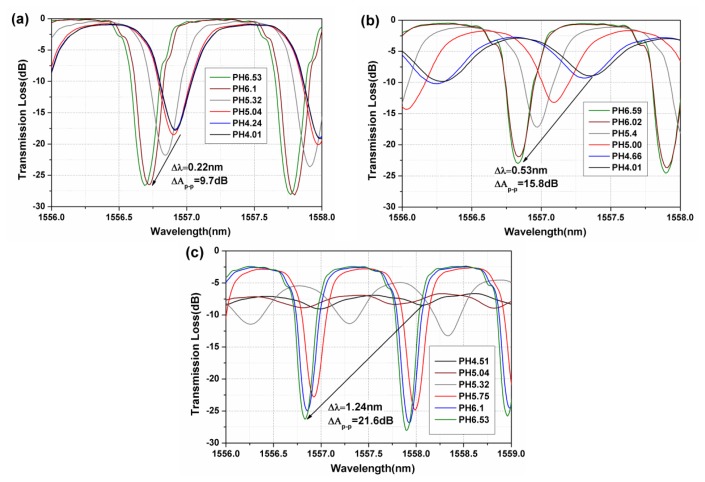
Evolution of optical spectra of TFBG pH sensors with different coatings [(***a***) (PDDA/PAA)_4_, (***b***) (PDDA/PAA)_6_, (***c***) (PDDA/PAA)_10_].

Figure 6 gives the spectral evolution of our fiber-optic sensors with pH for different thicknesses of sensing film. For each film, the pH of the aqueous solutions was adjusted by HCl or NaOH solution (in the range from 3.4 to 7.24). It can be seen that the pH increases, the coupling strength of the cladding mode resonances also increases. The reason is that with increasing pH, more carboxyl groups will be ionized and the sensing film becomes more hydrophilic. This in turn leads to swelling of the sensing film to allow more water inside it, and reduces the RI [3]. Due to the effective RI decrease of the sensing film the cladding modes become more strongly guided and less lossy, thereby recuperating some cumulative coupling strength along the grating As a result, the visibility of the fringe pattern observed in the transmission spectrum increases with the pH, a feature that will be used for quantitative analysis in a later section.

**Figure 7.**
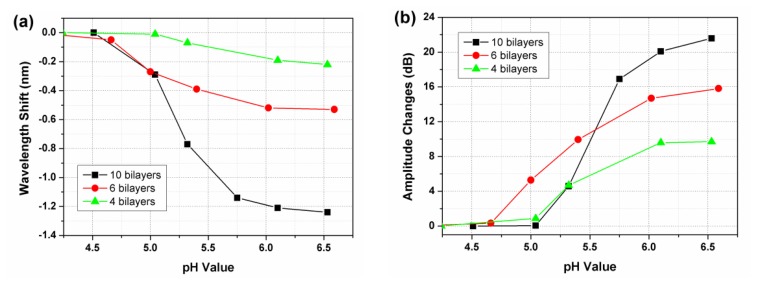
(***a***) Wavelength shift and (***b***) amplitude change of one resonance around 1,557 nm for three sensors with coatings having different numbers of bilayers (4, 6, 10).

Figure 7 shows the wavelength shift and amplitude changes of the resonance around 1,557 nm. For the sensors with 4, 6 and 10 bilayers of (PDDA/PAA) film, the wavelength shifts are 0.22, 0.53 and 1.24 nm, while the relative amplitude change is 9.7, 15.8 and 21.6 dB when the pH increases from 4.01 to 6.59. It is further observed that for certain ranges of the thickness of sensing film, the sensitivity (the slope of the curves in the Figures) also becomes larger.

For the three fiber-optic pH sensors that we prepared, the (PDDA/PAA)_10_ multilayer sensing film gives the highest overall sensitivity while the (PDDA/PAA)_4_ multilayer has the lowest one. This makes sense as the thicker initial coating has the highest effective RI, as felt by the evanescent field of the cladding mode, and hence the highest reaction to the uptake of water (with low RI) that comes about with the swelling [21]. We found the sensitivity of the sensor with (PDDA/PAA)_4_ to be too low because of the thinness of the sensitive coating, while the working range (linear response region) of the sensor with (PDDA/PAA)_10_ is too narrow.

The dynamic response of the pH sensor was also measured in the experiment. The MOI optical spectrum analyzer keeps sweeping with a sampling rate of 1 Hz. The response time (rise time) for the three fiber-optic sensors are 5, 10 and 30 s, respectively. It can be found that as the number of bilayers increases the response time becomes longer. Two reasons account for that. The longer diffusion time for water molecules from the outside of the film towards the fiber surface, and the increased density of thicker films (less porous as seen on Figure 2), which decreases the diffusion coefficient itself. For the films that we used, the best compromise for the time response and pH sensitivity, is the TFBG pH sensor with the (PDDA/PAA)_6_ coating.

**Figure 8.**
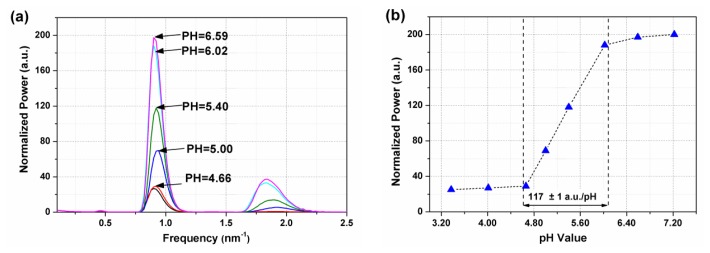
(***a***) Corresponding FFT spectra of TFBG pH sensor with (PDDA/PAA)_6_ coating. (***b***) The peak amplitude around 0.8 nm^−1^ changes with the pH of the solutions.

Since both wavelength shifts measurements and the determination of the “amplitude” of the oscillations in the spectrum are somewhat arbitrary, we now propose a non-equivocal quantitative measure of the effect of changing pH on the sensor response. To emphasize the visibility response of the sensor with (PDDA/PAA)_6_ coating, a band from 1,520 nm to 1,585 nm was selected for FFT analysis. Figure 8(a) depicts the results of this FFT analysis (using a Hanning window), while Figure 8(b) shows the amplitude of the dominant frequency peak around 0.8 nm^−1^ as a function of pH. One can see the response is linear for pH values in the range of 4.66 to 6.02. The obtained sensitivity is 117±1 a.u./pH unit, and with an accuracy of 0.01 pH. The response out of this range is anomalous, because from the low pH side, the effective RI of sensing polymer coating is close to that of water, while the high pH side, the effective RI is comparable to that of the fiber cladding. Both of two cases are out of the dynamic RI sensing range of TFBG itself (from 1.33 to 1.44 RIU) [22].

**Figure 9.**
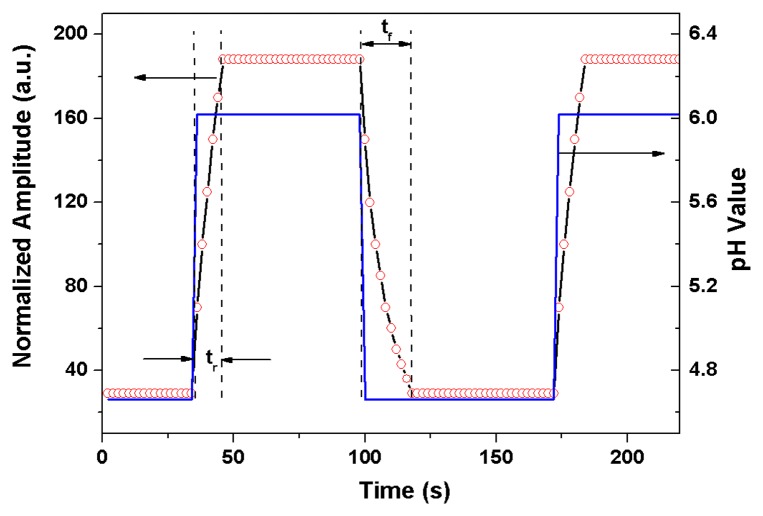
The measured dynamic response of the TFBG pH sensor with (PDDA/PAA)_6_.

For the dynamic response measurement, buffer solutions with pH values of 4.66 and 6.02 are alternately added into the testing groove. Figure 9 illustrates the measured dynamic response of the TFBG pH sensor with the (PDDA/PAA)_6_ coating, where the rise time (t_r_) of the sensor is 10 s and the fall time (t_f_) is 18 s. The response time for all our TFBG based sensors is shorter than the results of previous works [2,3,21,23]. As the AFM-measured surface morphology shows in Figure 2, these nanocoatings have high RMS so that they absorbs/desorbs the water efficiently and fast. Consequently, the swelling/shrinking process of the nanocoating exposed to solutions with different pH can be more efficient.

## 4. Conclusions

A polyelectrolyte sensing film has been successfully deposited on TFBG surfaces by the LbL electrostatic self-assembly technique to fabricate a novel fiber-optic pH sensor. The nanocoating was characterized by QCM and AFM. The main benefit of this kind of coating is its high RMS porosity, which makes the dynamic response very fast, of the order of 10 seconds. Our quantitative analysis technique for the sensor response yields a pH accuracy of 0.01, sufficient for many biomedical and process monitoring applications, especially given the fact that this miniature sensor (less than 1 cm long, with a 125 micrometer diameter) can easily be interrogated remotely. A disadvantage of this sensor is the limited linear operating range of pH (from 4.66 to 6.02) due to the high RI of the coating. We expect that other polyelectrolytes or hydrogels with lower RI can be mixed into the coating to reduce the effective RI and increase the measurement range. Thickness optimization is also in progress to obtain higher pH sensitivity.

## References

[1] Lemos S.G., Nogueira A.R.A., Torre-Neto A., Parra A., Alonso J. (2007). Soil calcium and pH monitoring sensor system. J. Agric. Food Chem..

[2] Gu B., Yin M.-J., Zhang A.P., Qian J.-W., He S. (2009). Low-cost high-performance fiber-optic pH sensor based on thin-core fiber modal interferometer. Opt. Express.

[3] Yin M., Gu B., Zhao Q., Qian J., Zhang A., An Q., He S. (2011). Highly sensitive and fast responsive fiber-optic modal interferometric pH sensor based on polyelectrolyte complex and polyelectrolyte self-assembled nanocoating. Anal. Bioanal. Chem..

[4] Yin M., Gu B., Qian J., Zhang A.P., An Q., He S. (2012). Highly sensitive and selective fiber-optic modal interferometric sensor for detecting trace mercury ion in aqueous solution. Anal. Methods.

[5] Michie W.C., Culshaw B., Konstantaki M., McKenzie I., Kelly S., Graham N.B., Moran C. (1995). Distributed pH and water detection using fiber-optic sensors and hydrogels. J. Lightwave Technol..

[6] Jones T.P., Porter M.D. (1988). Optical pH sensor based on the chemical modification of a porous polymer film. Anal. Chem..

[7] Wolfbeis O.S., Offenbacher H. (1986). Fluorescence sensor for monitoring ionic strength and physiological pH values. Sens. Actuators B..

[8] Hammond P.T. (2004). Form and function in multilayer assembly: New applications at the nanoscale. Adv. Mater..

[9] Decher G. (1997). Fuzzy nanoassemblies: Toward layered polymeric multicomposites. Science.

[10] Yin M., Qian J., An Q., Zhao Q., Gui Z., Li J. (2010). Polyelectrolyte layer-by-layer self-assembly at vibration condition and the pervaporation performance of assembly multilayer films in dehydration of isopropanol. J. Membr. Sci..

[11] Itano K., Choi J.Y., Rubner M.F. (2005). Mechanism of the pH-induced discontinuous swelling/deswelling transitions of poly(allylamine hydrochloride)-containing polyelectrolyte multilayer films. Macromolecules.

[12] Goicoechea J., Zamarreno C.R., Matias I.R., Arregui F.J. (2009). Utilization of white light interferometry in pH sensing applications by mean of the fabrication of nanostructured cavities. Sens. Actuators B..

[13] Corres J.M., Villar D.I., Matias I.R., Arregui F.J. (2007). Fiber-optic pH-sensors in long-period fiber gratings using electrostatic self-assembly. Opt. Lett..

[14] Shao L.-Y., Jiang Q., Albert J. (2010). Fiber optic pressure sensing with conforming elastomers. Appl. Opt..

[15] Zhang P., Qian J., An Q., Du B., Liu X., Zhao Q. (2008). Influences of solution property and charge density on the self-assembly behavior of water-insoluble polyelectrolyte sulfonated poly(sulphone) sodium salts. Langmuir.

[16] Zhao Q., Qian J., An Q., Gui Z., Jin H., Yin M. (2009). Pervaporation dehydration of isopropanol using homogeneous polyelectrolyte complex membranes of poly(diallyldimethylammonium chloride)/sodium carboxymethyl cellulose. J. Membr. Sci..

[17] Wang L.Y., Wang Z.Q., Zhang X., Shen J.C., Chi L.F., Fuchs H. (1997). A new approach for the fabrication of an alternating multilayer film of poly(4-vinylpyridine) and poly(acrylic acid) based on hydrogen bonding. Macromol. Rapid Commun..

[18] McAloney R.A., Sinyor M., Dudnik V., Goh M.C. (2001). Atomic force microscopy studies of salt effects on polyelectrolyte multilayer film morphology. Langmuir.

[19] Yang Y.-H., Haile M., Park Y.T., Malek F.A., Grunlan J.C. (2011). Super gas barrier of all-polymer multilayer thin films. Macromolecules.

[20] Burke S.E., Barrett C.J. (2004). pH-dependent loading and release behavior of small hydrophilic molecules in weak polyelectrolyte multilayer films. Macromolecules.

[21] Jesus M.C., Ignacio R.M., Ignacio D.V., Francisco J.A. (2007). Design of pH sensors in long-period fiber gratings using polymeric nanocoatings. Sens. J..

[22] Miao Y.-P., Liu B., Zhao Q.-D. (2009). Refractive index sensor based on measuring the transmission power of tilted fiber Bragg grating. Opt. Fiber Technol..

[23] Zamarreno C.R., Bravo J., Goicoechea J., Matias I.R., Arregui F.J. (2007). Response time enhancement of pH sensing films by means of hydrophilic nanostructured coatings. Sens. Actuators B..

